# Pathogenesis of the Homeostatic Failure of Ocular Surface as Morpho-Functional Unit

**DOI:** 10.3390/jcm11164685

**Published:** 2022-08-11

**Authors:** Antonio Di Zazzo

**Affiliations:** Ophthalmology Operative Complex Unit, University Campus Bio-Medico, 00128 Rome, Italy; a.dizazzo@unicampus.it

The ocular surface is a morpho-functional system in which multiple components such as cornea, conjunctiva, principal and accessory lacrimal glands, tear film, endocrine, immune, and nervous system cooperate to preserve local health. Innate, proportionate, and self-limiting immune response regulate the homeostatic equilibrium against daily repetitive insults and injuries. Once dysregulated, it may trigger overt inflammation, resulting in the clinical signs observed in several ocular chronic disorders. In such patients ocular surface system (OSS) dysfunction, possibly due to neurogenic, immune, metabolic and hormonal alterations leads to a critical shift in homeostatic baseline towards persistent inflammation, altered immune-responsiveness, and vulnerability to illness [1].

Ocular surface, as well as all body tissues, are continuously challenged by daily repetitive stimuli, injuries, and insults from the environment. Homeostatic physiologic balance is preserved by a strictly regulated inflammatory mechanism. This innate immune inflammatory response, also termed “parainflammation”, is self-limiting and directly proportioned to the insult intensity. Aggressive insults, such as infections or trauma, activate parainflammatory mechanisms, which help the tissue to restore its functionality and homeostasis. A protracted or disproportionate parainflammatory response may induce a shift of homeostatic set point towards inflammatory disease [2]. Parainflammation is a local low-grade subclinical immune reaction to harmful environments and helps tissue to adapt and maintain their adequate functionality. Practically parainflammation is a state between healthy homeostasis and chronic inflammation. A healthy immune system should initiate an effective parainflammatory response and control it [3]. In fact, such modulated and self-controlled immune innate parainflammatory response allows an ocular immuno and angiogenic privileged environment [4], in which inflammation resolves not merely because of the absence of pro-inflammatory signals, but rather by active inhibition pathways [5]; thus, regulating parainflammation is an active phenomenon simultaneously managed by nervous, endocrine, immune and vascular system [6,7,8,9]. Therefore, parainflammation is beneficial and dysregulated parainflammation is detrimental.

Dysregulation of parainflammatory mechanisms worsens into a “chronic abnormal inflammatory process” which leads to chronic detrimental immunologic events and various immune-related diseases, such as infection and autoimmune disorders. Low-grade chronic inflammation contributes to almost all age-related degenerative diseases, including those that occur in “immune privileged” tissues such as brain (Alzheimer and Parkinson diseases) and eye (age-related macular degeneration, dry eye disease), Cardiovascular system (obesity, atherosclerosis).

This Special Issue tries to investigate clinical and molecular inflammatory changes at the ocular surface in several ocular surface diseases in which such homeostatic mechanisms are dysregulated, showing persistent-chronic, subclinical, or excessive inflammatory response despite the insult.

Today, continuously increasing traffic, the ever-growing number of cars and trucks and their resultant fossil fuel emissions and airborne particulates, especially in big cities, along with global warming, have drastically modified air quality. Pollutants have been shown to directly initiate mucosal inflammation through several mechanisms, resulting in non-specific vasomotor conjunctivitis with allergic-like signs and symptoms, without an underlying apparent allergic mechanism and medical history of allergy [9].

This Urban Syndrome (US) causes an ocular surface inability to maintain its own homeostasis in response to non-specific insults. In such patients the ocular response appears excessively severe and unproportionate to environmental insult, leading to a system failure which cause red eye and ocular discomfort symptoms. Since US mostly affects young adults then, it critically impacts the occupational productivity.

Moreover, OSS is continually exposed to the environment and, consequently, to different types of microbes. This can occur during birth and at any time throughout life. It is possible that dysregulation of an ocular surface microbiotic community could trigger or contribute to any of these conditions There is mounting evidence to support the important role of the gut microbiome in the pathogenesis of ophthalmic diseases, suggesting it is a promising target for both preventive measures and therapeutic treatments. Changes in the immune system and metabolism, in turn, promoted the development of specific ophthalmic conditions in experimental settings [6,10].

The complexity of OSS immune system showed extensive involvement of innate and adaptive immunity components. Understanding the contribution of this immune diversity to the ocular surface is critical for ensuring successful local treatment as well as corneal allograft survival [2].

In addition to fighting a terrible disease, post-surgical breast cancer patients must face several systemic side effects induced by iatrogenic menopause and by estrogen deprivation therapy, as well as perioperative chemotherapy, with important psychological implications. Among these, Breast Cancer Iatrogenic Dryness (BCID) and specifically dry eye disease are life-threatening complications that critically affect women’s daily life, beyond their complex fighting against cancer. This status may be due to a loss or failure of immune regulatory parainflammatory mechanisms, which usually maintain homeostasis after hormonal changes, such as in other ocular diseases (Functional Androgenism, Polycystic Ovary Syndrome, Menopause). The BCID pathogenesis is also associated with a systemic estrogen-level drop, and a consequent functional hyperandrogenism, as in case of PCOS, and, rarely, with an unexpected hypoandrogenism, as in case of physiological menopause. Thus, these women after breast surgery experience a severe chronic evaporative dry eye syndrome with mucus filaments and frequent intolerance to wearing contact lenses. Most of them suffer from moderate itching associated with the usual ocular discomfort caused by the dryness [7].

Sex hormones may influence ocular surface-modulating immune response, leading to a low-grade subclinical inflammation, caused by the subverted para-inflammatory mechanisms that maintain ocular surface equilibrium. Therefore, an imbalance of sex hormones induces a dysregulation of the innate para-inflammatory response, which causes the failure of the ocular surface and overall mucosal dryness [7].

Breast cancer is the most common type of cancer among females, and it has devasting consequences in patients’ lives. Medical treatment and surgery related physical changes lead to a negative effect on one’s body image, depression and anxiety, as well as partner issues related to physical and hormonal changes. For many women, the consequences of iatrogenic menopause or estrogen deprivation therapy have the greatest negative impact on sexual function.

Breast cancer iatrogenic dryness is a systemic condition that is a consequence of the hormonal changes caused by necessary tumor treatments. The ocular involvement with severe dry eye, as well as the overall mucosal dryness, critically limits the daily life choice and activity of such women, therefore should be considered in the medical management of this large group of patients.

Considering the high quality of the articles submitted and published in this Special Issue, we want to thank all the authors for their precious contributions which will pave the way for new investigations in the field of ocular surface system failure. Further efforts of the scientific community, in conjunction with the valuable contributions and suggestions made by the authors of this Special Issue, are necessary to clarify the controversial point to identify dry eye disease as clinical picture caused by organ (ocular surface system) failure [11], maybe related to different pathogenesis. Does dry eye disease really exist?
